# Three-dimensionally reconfigurable focusing of laser by mechanically tunable metalens doublet with built-in holograms for alignment

**DOI:** 10.1515/nanoph-2022-0634

**Published:** 2023-01-16

**Authors:** Joonkyo Jung, Hyeonhee Kim, Jonghwa Shin

**Affiliations:** Department of Materials Science and Engineering, KAIST, Daejeon 34141, Republic of Korea

**Keywords:** alignment, hologram, metalens, multi-layer, varifocal

## Abstract

Metalenses have potential to replace various bulky conventional optical elements with ultrathin nanostructure arrays. In particular, active metalenses with reconfigurable focusing capability have attracted considerable interest from the academic and industrial communities. However, their tuning range is currently restricted by limited material properties and fabrication difficulties. Here, a hybrid optical system capable of three-dimensional relocation of a focal spot is proposed and experimentally demonstrated. The system comprises a mechanically actuated passive metalens doublet that can be easily fabricated with commonly available materials and processes. An incident laser can be focused to a desired point in three-dimensional space simply by rotating two metalenses or changing their separation. In addition, exploiting the polarization-multiplexing capability of metasurfaces, a hologram is incorporated to the metalenses to guide rotational and positional alignment of two metasurfaces. The ease of fabrication and alignment provided by this approach could widen its application to many practical fields.

## 1 Introduction

The advent of metasurfaces has triggered the era of flat optics [1]. Metasurfaces, which are planar arrays of intentionally designed nanostructures, have proven capability in controlling various properties of light such as its amplitude, polarization and phase within the thickness of subwavelength scale or even sub-nm scale [2]. In particular, dielectric metasurfaces have been widely studied because of their high efficiency, full 2π range of phase control, large degrees of freedom in polarization control and well-confined local response, which make them excellent candidates for a variety of phase and polarization-based devices such as lenses [3–6], holograms [7, 8], vector beam generation [9, 10], and other novel applications. Furthermore, it was recently demonstrated that a single metasurface with closely stacked bi-layer structures affords complete linear control of light transmission [11]. This kind of dielectric metasurface also has the advantage of relatively easy fabrication; conventional silicon lithographic processes can be used to fabricate metasurfaces with high fidelity.

On top of these successful demonstrations of passive metasurfaces, researchers have sought to implement metasurfaces with reconfigurable or real-time tunable properties, for applications such as dynamic holography [12, 13], tunable beam-steering [14, 15], spectropolarimetry [16], and color switching [17]. By changing the properties of the constituent material or by manipulating the environment surrounding the metasurfaces, the amplitude, phase, and polarization responses of metasurfaces can be actively controlled. Thus far, researchers have proposed many different approaches as the tuning mechanism, including electrical tuning by applying voltage [14, 15], thermal tuning based on phase-change material [18, 19], micro-electro-mechanical systems (MEMS) [20–24], deformable substrates [25, 26], and mechanical tuning of the configuration [22], [23], [24, 27], [28], [29]. Typically, MEMS, electrical and thermal tuning mechanisms can modify the metasurfaces in a pixel-by-pixel manner. This allows very large degrees of freedom and the metasurface can transform from a convex lens to a concave lens, grating, hologram, or any combinations thereof. However, in order to achieve high numerical apertures for lenses, large diffraction angles for gratings, or large viewing angles for holograms, the size of independently tunable pixels should be comparable to the wavelength or even smaller, which may prohibit utilization of MEMS in such applications. Even for local voltage or temperature tuning with no moving parts, this condition necessitates densely packed feed lines and presents challenges in terms of fabrication, especially for visible or near-infrared wavelength regimes. To reduce the required complexity of fabrication, their controllability is generally restricted to low resolution (large pixel size) or one-dimensional control.

Deformable substrates or mechanical realignment of multiple metasurfaces can provide alternative pathways towards tunable optical systems based on metasurfaces. Since nanostructures constituting the metasurfaces do not have to be controlled in a pixel-by-pixel manner in these approaches, they can be easily applied to visible and near-infrared wavelength regimes as well as terahertz or microwave regimes. Tunable metalenses based on deformable substrates have been demonstrated for controlling the focal length [25, 26] or the focal position [25] by stretching the substrates. However, their response time is currently limited to millisecond scale or longer because of elastomer’s viscoelasticity [25]. Mechanical reconfiguration of positions or orientations of multiple metasurfaces has also been studied for varifocal lens [22], [23], [24, 29] and beam shaping [27, 28, 30]. In this mechanism, by tuning the separation between two metasurfaces [22, 23] or the rotational angles of two metasurfaces [27–30], the devices can be actively tuned to realize different properties or functions. The ease of fabrication of these classes of nanostructure-based tunable optical devices makes them a good candidate for practical applications. However, the relative lack of degrees of control freedom for deformable substrates and the issue of precise positional and angular alignment of multiple metasurfaces are remaining hurdles.

One of the most powerful strengths of metasurfaces, compared to conventional refractive optics, is that they can be designed to have multiplexed functionalities for different input polarization states [31]. So-called polarization-multiplexed metasurfaces allow two totally different devices to be embodied within a single metasurface. Since polarization-multiplexing naturally arises from the anisotropy of nanostructures, any additional fabrication complexity is not required. Several studies have demonstrated a bifocal metalens based on this polarization-multiplexing technique [32, 33].

Here, we present a tunable focusing device with a three-dimensionally relocatable hot spot based on mechanical tuning of metalens doublet. Two constituting metasurfaces function as beam-steering convex and concave lenses, respectively. Their rotational angles determine transversal location of the hot spot and the distance between them controls the effective focal length of the doublet and, hence, the longitudinal position of the high intensity point. With analytic derivation, we present a closed-form expression of the location of the focal spot in three-dimensional space. The experimentally measured beam shapes show good agreement with the predicted behavior. The fabrication error tolerance of metasurfaces is also discussed to support the practicality of our metalens doublet scheme. In addition, making use of the polarization-multiplexing capability of metasurfaces, we demonstrate that the metalens doublet can work as a variable focal-spot device for right-circularly polarized (RCP) light and generate a visual alignment-guiding hologram for left-circularly polarized (LCP) light. This auxiliary hologram can help users check and maintain the precise alignment of two metasurfaces in real-time without disrupting the variable focal-spot operation.

## 2 Results and discussion

### 2.1 Principle of reconfigurable focus

**Figure 1:**
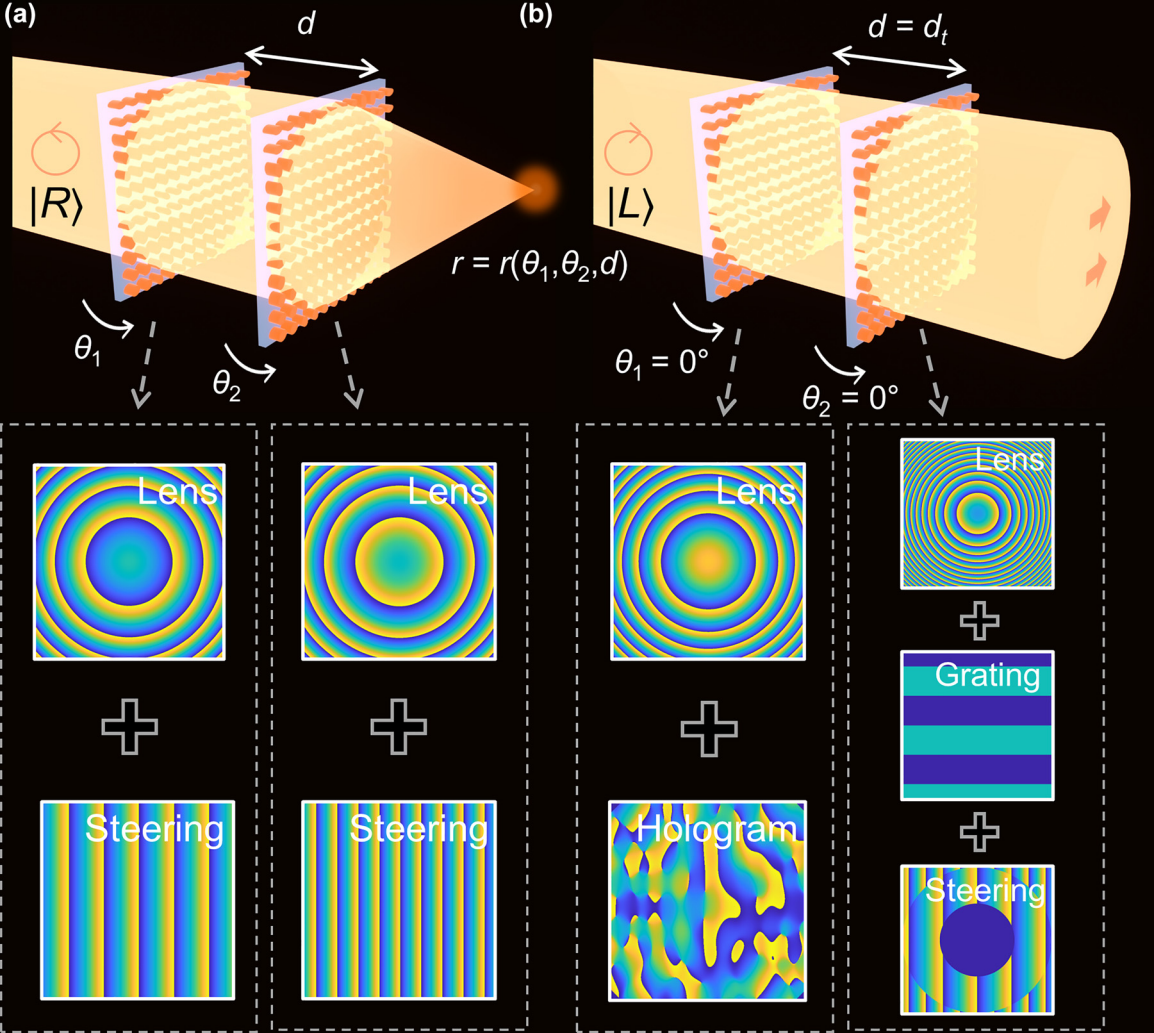
Tunable metalens doublet with built-in hologram for alignment. (a) and (b) Schematic illustration of two different functionalities depending on input polarization states. The bottom panel shows the constituting phase pattern for each layer and each polarization state. (a) Tunable metalens doublet under RCP input polarization state. The position of the focus is determined by rotation angles of each layer (*θ*_1_,*θ*_2_) and interlayer distance *d*. (b) Built-in hologram for alignment under LCP input polarization state. The holographic image reveals the current angular and positional alignments of two metasurfaces.

**Figure 2:**
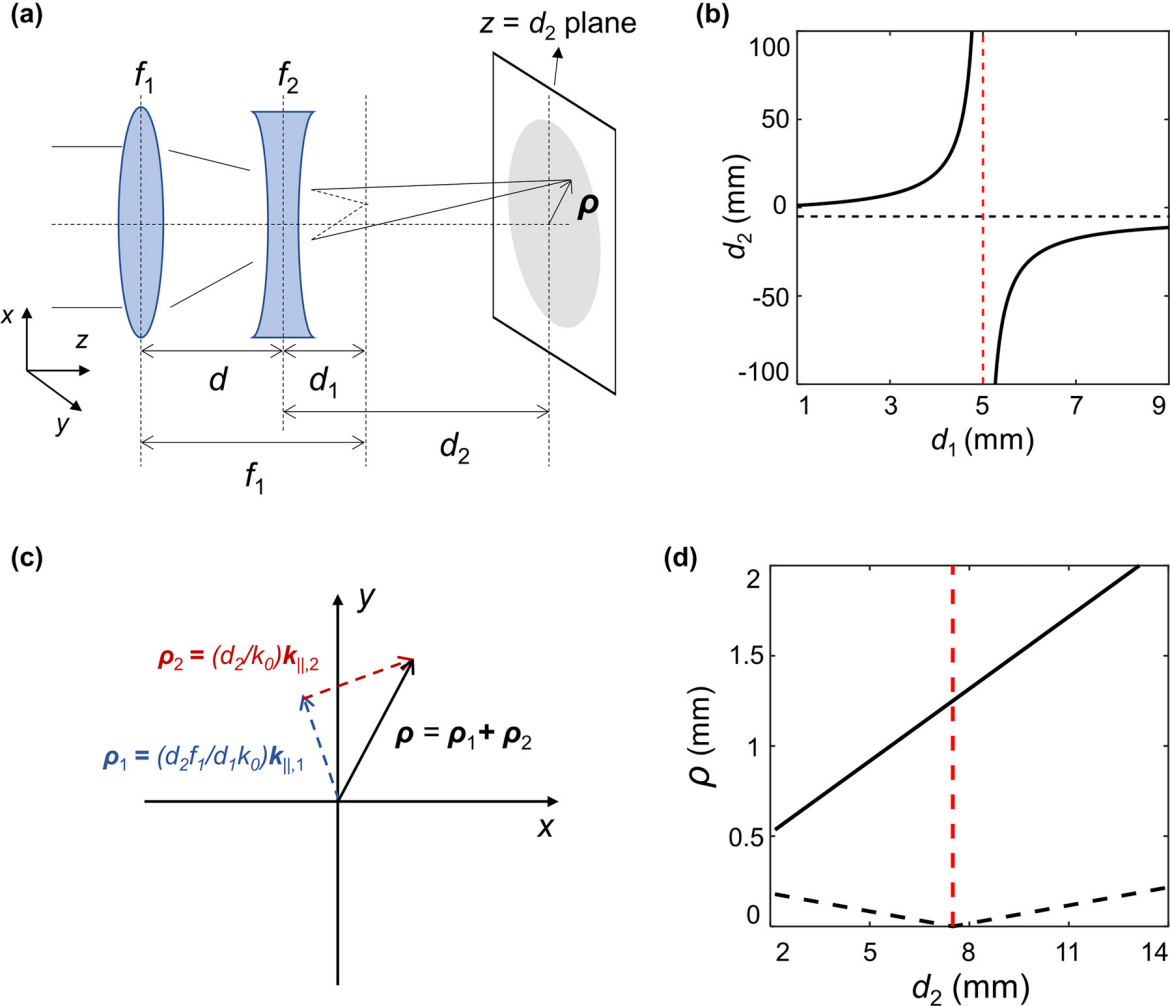
Principle of reconfigurable hot spot. (a) Schematic description of the reconfigurable focusing device. It consists of two beam-steering convex and concave lenses. (b) Relation between *d*_1_ and *d*_2_ based on the imaging equation. (c) Vector sum representation of Eq. (1). (d) Allowed displacement of the hot spot from the optical axis. The black solid (dashed) line shows the maximal (minimal) transversal displacement as a function of *d*_2_. The red line represents the condition at which the minimal displacement is zero.

The tunable metalens doublet consists of two optical elements, each of which is functionally a superposition of a beam-steerer and a lens, or, equivalently, a decentered lens [30] as described in Figure 1(a) (For LCP, the doublet forms an alignment-dependent far-field hologram as shown in Figure 1(b), which will be explained in Section 2.5). Figure 2(a) shows a schematic of the working principle. Throughout the paper, the position of the second lens is set to be the origin (*z* = 0). In this scheme, focal lengths of the first and second elements (*f*_1_ and *f*_2_, respectively) and their separation determines the effective focal length of the whole device. The first convex lens focuses a collimated input beam at a distance *f*_1_ from the first lens, and the second concave lens moves the longitudinal position of the hot spot to *z* = *d*_2_ according to the imaging equation given as $\frac{1}{-d_{1}}+\frac{1}{d_{2}}=\frac{1}{f_{2}}$ . *d*_1_ is the difference between *f*_1_ and the separation of the two metasurfaces, and the signs imply that the object is virtual and the image is real. Note that the simple ray-optics lens formula can be applied here because the Rayleigh lengths of the incident beams to the first and second lenses are much larger or much smaller than the focal lengths of the metalenses [34]. Figure 2(b) shows the value of *d*_2_ as a function of the separation of two elements. Since the effective focal length is dependent on the separation between lenses, one can control the effective focal length dynamically by manipulating the separation.

Furthermore, the beam-steering effect of the first and second lens determines the transversal displacement ***ρ*** of the hot spot. Beam steering can be quantified with an additional tangential wavevector ***k***_‖_ imparted by the element. If there are two beam steerers without a lensing effect, the combined effect can be expressed as the vector sum of the two tangential wavevectors of each element in the spatial frequency domain [28]. However, in the proposed scheme, because of the lensing effect, the combined effect is not a simple vector sum of such vectors and the object and image locations and the focal length must be considered as well. We derived the transverse position of the focal spot using the Fresnel approximation (see Supplementary Materials, section S1 for the detailed derivation). At the imaging plane, the transversal displacement effect of metalens doublet is given as

(1) $$\begin{array}{ll} \rho & =\frac{d_{2}}{k_{0}}\left(k_{\|,2}+\frac{f_{1}}{d_{1}}k_{\|,1}\right)\\ & =\frac{d_{2}}{k_{0}}\left[\begin{array}{c} \left|k_{\|,2}\right|\cos\theta_{2}+\frac{f_{1}}{d_{1}}\left|k_{\|,1}\right|\cos\theta_{1}\\ \left|k_{\|,2}\right|\sin\theta_{2}+\frac{f_{1}}{d_{1}}\left|k_{\|,1}\right|\sin\theta_{1} \end{array}\right] \end{array}$$

where *k*_0_ is a wavenumber in free space, $k_{\|,i}\left(i=1,2\right)$ , the beam-steering tangential wavevector of *i*th element and $\theta_{i}\left(i=1,2\right)$ , the rotation angle of the *i*th element. Note that this is a vector sum of tangential wavevectors with weighting factors as described in Figure 2(c). From the expression, it is clear that one can control the transversal displacement by simply manipulating two rotation angles. Consequently, by combining both mechanical degrees of freedom of the separation and the rotation angles, the focus of the metalens doublet can be arbitrarily relocated in three dimensions within the range allowed by specific choices of the focal lengths and the steering wavevectors. We note that there is a minimum radius within which the hot spot can be placed; if the second element is designed to have a tangential wavevector satisfying $\left|k_{\|,2}\right|=\frac{f_{1}}{d_{1,t}}\left|k_{\|,1}\right|$ , where *d*_1,t_ is a target distance, the minimum radius reduces to zero if *d*_1_ = *d*_1,t_. At other distances, the minimum radius is non-zero. In general, the achievable region with an arbitrary positive *d*_1_ can be analytically obtained from Eq. (2) and is given as

(2) $$\begin{array}{c} \rho_{\pm }=\frac{d_{2}f_{1}}{k_{0}}\left|k_{\|,1}\right|\left|\frac{1}{d_{1,t}}\pm \frac{1}{d_{1}}\right| \end{array}$$

where *ρ*_+_(*ρ*_−_) is the maximum(minimum) value of transversal displacement. Figure 2(d) presents the calculated maximum and minimum displacement as a function of *d*_2_. As can be seen in Figure 2(d), when *d*_1_ ≠ *d*_1,t_, the achievable region has a doughnut-like shape and the central region is inaccessible. (a three-dimensional illustration of the achievable sweeping region is also given in Supplementary Materials, Figure S2). In addition, since *d*_1_ → −*f*_2_ as *d*_2_ → ∞, the maximal and minimal displacements in Eq. (2) are approximated as $\rho_{\pm }\approx \frac{d_{2}f_{1}}{k_{0}}\left|k_{\|,1}\right|\left(\frac{1}{d_{1,t}}\mp \frac{1}{f_{2}}\right)$ in this regime of large *d*_2_. Therefore, the proposed metalens doublet can be regarded as a varifocal lens with variable steering angle where the maximal and minimal angles (*θ*_+_ and *θ*_−_) are given as $\theta_{\pm }=\tan^{-1}\left(\frac{\rho_{\pm }}{d_{2}}\right)\approx \tan^{-1}\left(\frac{f_{1}}{k_{0}}\left|k_{\|,1}\right|\left(\frac{1}{d_{1,t}}\mp \frac{1}{f_{2}}\right)\right)$ . If the application requires full access to a contiguous volume without any hole, one can limit the scan region to a half of the doughnut. If required, the entire system can be tilted with respect to the optical axis such that such uninterrupted region is centered around the optical axis (see Supplementary Materials, section S2).

### 2.2 Design and fabrication of metasurface

**Figure 3:**
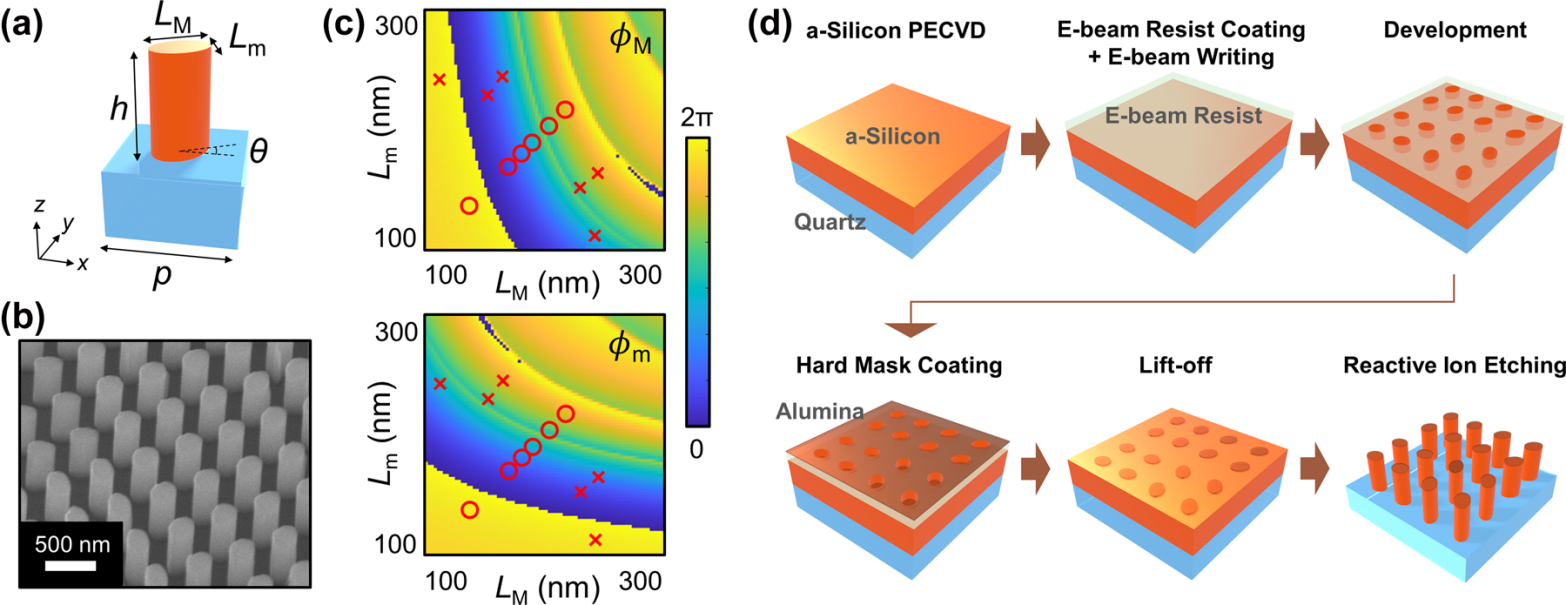
Fabrication of metasurface. (a) Schematic of a unit cell with an elliptical nanopost. (b) SEM image of fabricated sample. *p*: period; *h*: height; *L*_M(m)_: length of the major (minor) axis; *θ*: orientation angle of the major axis with respect to the *x*-axis. (c) Simulated phase accumulation along the principal axes. The red circles (crosses) correspond to the selected PI (PS) structures listed in Table 1. *Φ*_M(m)_: phase accumulation along the major (minor) axis. (d) Fabrication processes for the metasurfaces.

Metasurfaces are composed of spatially-varying nanostructures. One should design the structural parameters of nanostructures at a specific position based on the local response of the metasurface required at that position. If nanostructures have anisotropic shapes, the resulting responses also become anisotropic or polarization-sensitive (PS) and the response depends on the input polarization states. We adopt an elliptical nanopost as an anisotropic unit structure of the metasurfaces as depicted in Figure 3(a). Figure 3(b) shows a scanning electron microscopy (SEM) image of the fabricated sample. For highly transmissive dielectric metasurfaces, this anisotropic response can be approximated well using a unitary symmetric Jones matrix [31, 35], which is given as

(3) $$\begin{array}{c} U_{s}=\left[\begin{array}{cc} \cos\theta & -\sin\theta \\ \sin\theta & \cos\theta \end{array}\right]\left[\begin{array}{cc} {\text{e}}^{\text{i}\phi_{\text{M}}} & 0\\ 0 & {\text{e}}^{\text{i}\phi_{\text{m}}} \end{array}\right]\left[\begin{array}{cc} \cos\theta & \sin\theta \\ -\sin\theta & \cos\theta \end{array}\right] \end{array}$$

where *ϕ*_M_ and *ϕ*_m_ are phase accumulation of the principal directions obtained while passing through the nanopost and *θ* is the rotation angle of the structure. The phase responses *ϕ*_M_ and *ϕ*_m_ are directly related to the structural parameters *L*_M_ and *L*_m_ of a unit structure (Figure 3(a)). To relate (*ϕ*_M_, *ϕ*_m_) to (*L*_M_, *L*_m_), finite-difference time-domain (FDTD) simulations were employed (see Methods section for details). For the sake of experimental feasibility, parameters ranging from 100 nm to 300 nm were explored for *L*_M_ and *L*_m_ given a unit cell period of 500 nm. The obtained phase accumulations are shown in Figure 3(c) (amplitudes of transmitted light are also given in Supplementary Materials, Figure S4).

Metasurfaces can be designed to be dependent or independent of input polarization states. Both cases require different categories of nanostructures. For polarization-insensitive (PI) devices, the phase accumulations of principal axes should be the same (*ϕ*_m_ = *ϕ*_M_). For that purpose, nanostructures with C4 symmetric cross-sections where *L*_M_ and *L*_m_ are identical are required. For PS devices, we chose RCP and LCP as input polarization states because of its rotational symmetry. In this case, nanostructures with the phase accumulations satisfying *ϕ*_m_ = *ϕ*_M _+ π are required (see Methods section for the details). Note that the handedness of the input polarization states is flipped after passing through PS metasurfaces. We selected two groups of nanostructures for PI and PS designs, which are marked in Figure 3(c) and listed in Table 1. Each group consists of six different structures corresponding to six equally distributed phase accumulations of $\phi_{\text{M}}=\left\{\frac{3\pi }{12},\frac{7\pi }{12},\frac{11\pi }{12},\frac{15\pi }{12},\frac{19\pi }{12},\frac{23\pi }{12}\right\}$ . With the selected structural parameters organized in the appropriate position, the desired phase map can be approximately implemented with the given six-level phase modulation.

**Table 1:** Structure library for PS and PI design.

Structure no.	Phase (*ϕ*_M_, *ϕ*_m_)	Dimension (*L*_M_, *L*_m_)
PS #1	(3π/12, 15π/12)	(152.5 nm, 230 nm)
PS #2	(7π/12, 19π/12)	(165 nm, 245 nm)
PS #3	(11π/12, 23π/12)	(242.5 nm, 112.5 nm)
PS #4	(15π/12, 3π/12)	(230 nm, 152 nm)
PS #5	(19π/12, 7π/12)	(245 nm, 165 nm)
PS #6	(23π/12, 11π/12)	(112.5 nm, 242.5 nm)
PI #1	(3π/12, 3π/12)	(170 nm, 170 nm)
PI #2	(7π/12, 3π/12)	(181 nm, 181 nm)
PI #3	(11π/12, 3π/12)	(190 nm, 190 nm)
PI #4	(15π/12, 15π/12)	(204 nm, 204 nm)
PI #5	(19π/12, 19π/12)	(217.5 nm, 217.5 nm)
PI #6	(23π/12, 23π/12)	(137.5 nm, 137.5 nm)

Figure 3(d) describes the detailed fabrication process. Amorphous silicon (thickness of 790 nm) is deposited on a quartz glass substrate by plasma-enhanced chemical vapor deposition (PECVD). An adhesion layer (AR 300-80, Allresist), electron-beam resist (AR-P 6200.04, Allresist), and conductive polymer (AR-PC 5090.02, Allresist) are then uniformly spin-coated consecutively for electron-beam lithography. Designed structural parameters are patterned by an electron-beam writing process with a dose level of 4.2 C/m^2^ and developed. An alumina hard mask (60 nm) is deposited using electron-beam evaporation and residual resist is lifted off for the etching step. To realize high aspect ratios with vertical side walls, deep reactive ion etching (RIE) was employed (pseudo-Bosch dry etching). The etching condition was thoroughly calibrated as it is the most critical step affecting the shape of the nanostructures and, as a result, device performance. For this purpose, not only the gas fraction but also the over-etching time and amount of thermal paste, which can affect the final sidewall slope, are carefully tuned. For etching, the gas flows of C_4_F_8_ and SF_6_, chamber pressure, coil power, and platen power are set to be 43.5 sccm (C_4_F_8_), 16.5 sccm (SF_6_), 25 mTorr, 600 W, and 30 W, respectively.

### 2.3 Analysis on tolerance for fabrication errors

During the fabrication process, fabrication errors are inevitable. For the described fabrication process, the three main factors affecting the final shape of nanostructures are inaccuracy in pattern size, layer thickness, and sidewall slope. In this section, we analyzed how much these errors affect the optical properties of the selected 12 nanopost structures and the final devices using FDTD simulations and numerical calculations based on Fourier optics. Only the results for PS design are shown in the manuscript (see Supplementary Materials, Figure S5 for the results of PI design).

**Figure 4:**
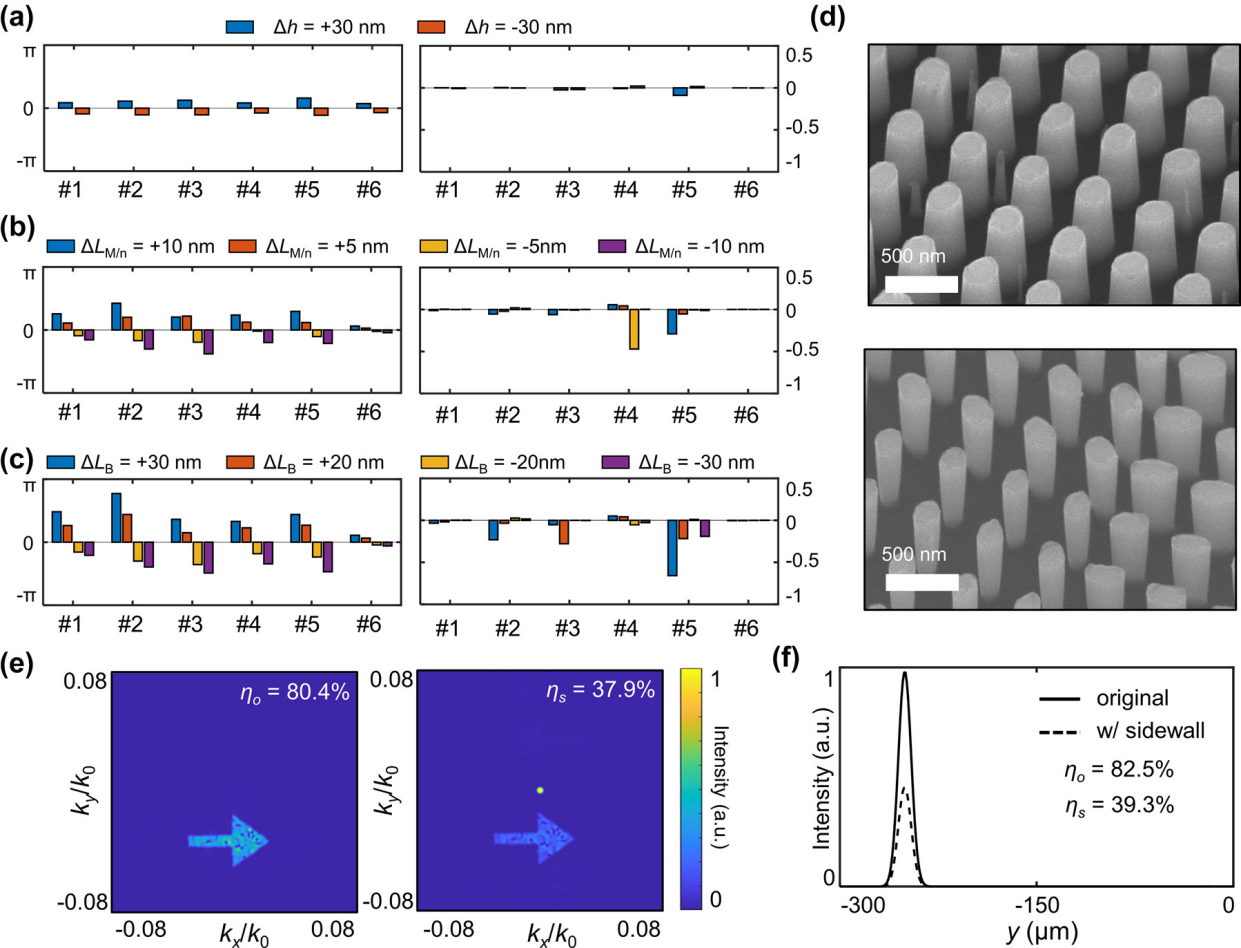
Analysis on fabrication error. (a–c) Phase (left panels) and amplitude (right panels) deviations from the target values for the selected structures of the PS design when there are errors in (a) thickness, Δ*h*, (b) pattern size along the major and minor axes, Δ*L*_M/m_, and (c) sidewall slope. (The bottom cross-section deviates from the top cross-section by Δ*L*_B_, if the sidewall is not perfectly vertical.) (d) SEM image of fabricated samples with positive (top panel) and negative (bottom panel) sidewalls. (e) and (f) Performance of devices consisting of unit cells with and without fabrication error. The fabrication error is set to be Δ*L*_B_ = 30 nm. (e) Hologram. *η*_o(s)_: power efficiency of the device without (*η*_o_) and with (*η*_s_) the error. (f) Beam-steering lens. *η*_o(s)_: power efficiency of the device without (*η*_o_) and with (*η*_s_) the error.

Errors in thickness of the nanopost occur during deposition of the silicon layer. Figure 4(a) shows the phase and amplitude deviation from the target value for the selected structures with thickness deviations of ±30 nm for PS design. In general, thicker structures cause positive phase deviation and thinner structures cause negative phase deviation. From the view point of interpreting the nanopost as a waveguide [31], it can be intuitively understood that longer waveguides give larger phase accumulations. On the other hand, amplitude discrepancies are small.

Errors in pattern size usually occur during the electron beam writing process, mainly due to the proximity effect [36]. Figure 4(b) shows the phase and amplitude deviation from the target value for the selected structures with inaccurate pattern size of ±5 nm and ±10 nm from the ideal design. Interestingly, there is a tendency that positive errors in pattern size cause positive phase deviation, and negative errors cause negative phase deviation. In addition, the magnitude of the phase deviation becomes larger as errors in pattern size become larger. On the contrary, each structure shows different tendencies with respect to amplitude deviation. For example, the fourth(fifth) structure shows large amplitude deviation when the pattern size is smaller(larger) than the designed dimension. These phenomena are attributed to a resonance dip in transmission of the nanopost structures. As the dimension of nanostructure changes because of inaccurate pattern sizes, the resonance dip in transmission moves as well because the resonance wavelength is roughly proportional to the physical size of the resonator. Depending on the relative position of resonances compared to the target wavelength of 915 nm, each nanostructure experiences different dependency on errors in pattern size: if the resonance wavelength is far from 915 nm, that resonance does not substantially affect the transmission at the target wavelength; if the resonance wavelength is slightly smaller than the target, its shift affects transmission amplitude strongly for positive error in pattern size and does not affect significantly for negative error, if the resonance wavelength is slightly larger than the target, vice versa (see Supplementary Materials, section S3 for a detailed analysis).

Errors in sidewall generally occur during the etching process. Figure 4(c) describes the phase and amplitude deviation from the target value for the selected structures with sidewall slopes making bottom ellipses deviate in size by amounts of ±20 nm and ±30 nm compared to the top ellipses with the ideal dimensions. Figure 4(d) presents SEM images of the fabricated samples with positive and negative sidewall slopes. Note that a vertical sidewall requires strict process conditions; the above samples were obtained when the gas flows varied 1 sccm from the correct condition and thermal paste was not perfectly spread. Phase and amplitude deviations show a similar tendency to the case of errors in pattern size. If the sidewall slope is positive, the phase deviation is positive, and if the sidewall slope is negative, then the phase deviation is also negative. Similar structures show a similar tendency in amplitude deviation, for example, the fifth structure show a large amplitude deviation for positive errors in the sidewall slope, as in the case of a positive error in pattern size.

To quantitatively evaluate the effect of inaccurate fabrication on a device level, we designed two phase-based devices; a simple hologram and a lens (see Methods for the details). To compare the performance of devices with and without fabrication errors, we numerically calculated the performance of the devices with ideal parameters and the devices with intentionally introduced fabrication errors. Since errors in thickness, pattern size, and sidewall slope tend to be globally similar, we assumed all the structures over the entire devices have the same fabrication error for the calculations. As in Figure 4(e) and (f), despite the phase and amplitude deviations of individual structures, the overall functions of the metasurfaces can be maintained albeit with lower efficiency. For the hologram, the efficiency dropped from 80.4% to 37.9%, with the degraded holographic image. For the lens, the efficiency dropped from 82.5% to 39.3%. Note that these efficiency drops are the worst cases with +30 nm sidewall error (see Supplementary Materials, Table S1 for the list of efficiencies with other fabrication errors).

### 2.4 Tunable metalens doublet

For a proof of concept, we first designed and fabricated metalens doublet targeting 915 nm wavelength for both PS and PI designs. Only the results of the PS design are shown in the manuscript (see Supplementary Materials, Figure S7 for the results of the PI design). As illustrated in Figure 1(a), for the tunable metalens doublet, the first metalens is designed to perform as a beam-steering convex lens and the second metalens is designed to perform as a beam-steering concave lens (see Methods for details). Taking experimental feasibility into consideration, the selected lens parameters are as follows: *f*_1_ = 5 mm, $\left|k_{\|,1}\right|=0.05k_{0}$ , *f*_2_ = −5 mm, *d*_1,t_ = 3 mm, and $\left|k_{\|,2}\right|=\frac{f_{1}}{d_{1,t}}\left|k_{\|,1}\right|=0.0833k_{0}$ (see Methods for the design details of metasurfaces). The designed metasurfaces were fabricated following the fabrication process described in the previous section. Note that since PS metasurfaces flipped handedness of input polarization states, the output polarization states after passing through doublet are the same as the input polarization states (see Supplementary Materials, section S4 for the details).

**Figure 5:**
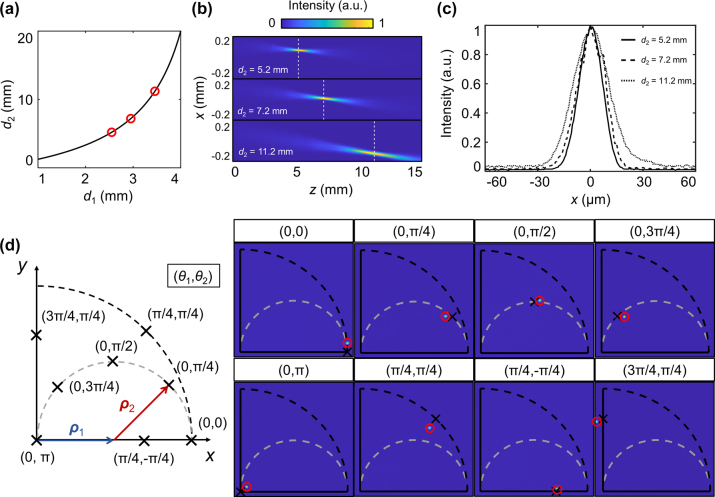
Reconfigurable focusing by tunable metalens doublet. (a)–(c) Longitudinal controllability of the hot spot position (a) Relation between *d*_1_ and *d*_2_. The black line corresponds to the relation from the imaging equation. The red circles represent (*d*_1_, *d*_2_) = {(2.6 mm, 5.2 mm), (3 mm, 7.2 mm), and (3.5 mm, 11.2 mm)}, which are obtained from numerical calculations based on Fourier optics. (b) Numerically calculated field profiles. The white dashed lines show expected *d*_2_ for each case. (c) Experimentally measured intensities at different *d*_2_ values. (d) Transversal controllability of the hot spot position. The left panel shows target positions for different combinations of rotation angles of two metasurfaces. The right panels show the measured field profiles. The measured hot spot is on the center of red circle.

To validate the fabricated metalens doublet, we imaged the focused beam with various combinations of separation and rotation angles of the metasurfaces (see Methods and Supplementary Materials, Figure S9 for a detailed description of the measurement setup). Figure 5(a)–(c) prove the longitudinal controllability of the hot spot. We measured the effective foci at three different *d*_2_ positions marked in Figure 5(a) with appropriate separation for each case. Note that there is small difference between theoretically expected *d*_2_ (5.4 mm, 7.5 mm, and 11.6 mm, respectively) and experimentally measured *d*_2_ (5.2 mm, 7.2 mm, and 11.2 mm, respectively). This difference is mainly due to the discrepancy between the Fresnel approximation and actual beam propagation. In Figure 5(b), numerically calculated field profiles with *θ*_1_ = 0 and *θ*_1_ = π assumed along the beam propagation direction are shown. Numerical calculations were performed based on Fourier optics. The field profiles support the measured *d*_2_ values. The measured field profiles at different *d*_2_ values are shown in Figure 5(c). Their full width at half maximum (FWHM) is 15.2 μm, 17.8 μm, and 22.9 μm, respectively, which is similar with the calculated result of 16 μm, 18 μm, and 24 μm, respectively, given an incidence beam with a waist radius of 250 μm. The transversal controllability of the hot spot can be checked in Figure 5(d). The left panel shows the expected hot spot positions on the image plane for different combinations of the rotation angles of two metalenses. Dashed lines are the calculated trajectories of the hot spot as *θ*_2_ changes while *θ*_1_ is fixed to zero (gray line) and as *θ*_1_ and *θ*_2_ increase by the same amounts (black line), respectively. Note that two vectors (***ρ***_1_ and ***ρ***_2_) have the same magnitude because *d*_1_ = *d*_1,t_. The measured intensity pattern for combinations of rotation angles are shown in the right panel of Figure 5(d). The calculated and measured focal positions are well matched.

The designed metalens doublet are robust against monochromatic aberrations such as spherical aberration, coma, astigmatism and Petzval field curvature because the sample size is much smaller than the focal length and the incidence angle to the second metalens is small [37]. On the contrary, the doublet suffers from chromatic aberration. The chromatic aberration makes each metalens have a different focal length and steering angle when the input wavelength is different from the target wavelength [38]. This chromatic aberration can be improved further, if needed, by considering not only phase but also its dispersion when designing nanoposts as in [3–6].

### 2.5 Visual alignment-guiding hologram

To maximally utilize the potential of metasurfaces, we designed a PS metalens doublet with polarization-multiplexed functionalities; for the RCP input, the device performs as a metalens doublet, and for the LCP input, the same device performs as a built-in hologram for alignment as described in Figure 1(a) and (b). Note that this hologram is only one of numerous examples of new functionalities that metasurfaces can support.

**Figure 6:**
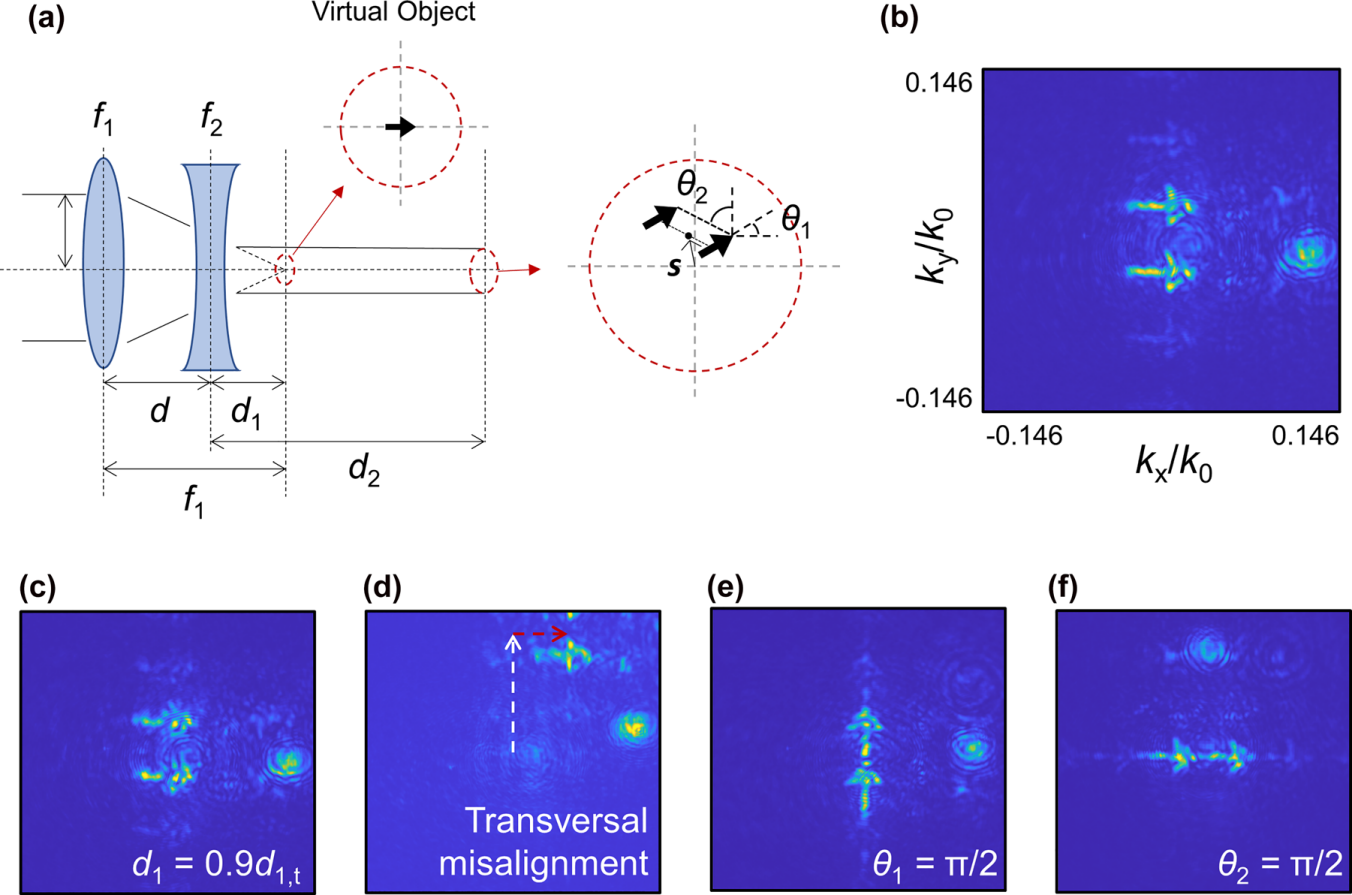
Built-in hologram for alignment. (a) Schematic illustration of operation principle of built-in holograms. *θ*_1(2)_: the rotation angle of the first (second) metasurface; ***s***: displacement vector due to misalignment. (b) Measured holographic image when the metasurfaces are well aligned. (c)–(f) Measured holographic images with misalignment. (c) With longitudinal misalignment. (d) With transversal misalignment along the vertical direction. White arrow shows the intrinsic displacement caused by lens characteristics. Red arrow shows the extrinsic displacement caused by spatially-varying beam-steering effect. (e) With the first metasurface rotated by 90°. (f) With the second metasurface rotated by 90°.

Figure 6(a) describes the working principle of the alignment-guiding hologram. For the LCP input, the first metasurface is designed to perform as a superposed device of a hologram generating a holographic image of an arrow and a convex lens with a focal length *f*_1_ = 3.5 mm. Without the second metasurface, it simply generates the focused holographic image at the image plane. The second metasurface is designed to perform as a triply superposed device of a concave lens with a focal length *f*_2_ = −1.5 mm, a simple grating with a period Λ = 40 μm along the vertical direction, and a spatially-varying beam-steerer, as in Figure 1(b) (see Methods for the detailed design). The three functionalities of the second metasurface fulfill the following roles: the concave lens transfers the focused arrow image of the first metasurface acting as a virtual object to the far-field regime; the simple grating makes an array of the copied arrow images along the grating direction; and the spatially-varying beam-steerer discretely shifts the images in the lateral direction corresponding to its gradient of phase at the position where the beam is incident. As a whole, the resulting holographic image contains all the information about state of alignment of two constituting metasurfaces and can be readily measured without direct imaging of the metasurfaces. First, from the direction of the arrow, the rotation angle of the first metasurface can be identified since the arrow image is designed to indicate the positive *x*-direction. Second, from the direction of separation of the two arrows, the rotation angle of the second metasurface can be identified since the separation is originated from the grating phase, which is originally aligned along the positive *y*-direction of the second metasurface. Third, the distance between two metasurfaces is directly related to the clarity of the image. Since both metasurfaces have lens phases, if the separation between them is incorrect, the output beam becomes diverging rather than collimated, and as a result, the resulting image becomes blurred. Hence, the clearer the image is, the more accurate the longitudinal alignment will be. Fourth, transversal alignment is related to the position of the center of twin arrows. Basically, the position of the center of twin arrows is moved when two metasurfaces are misaligned because of their lens phases, as in the case of two misaligned lenses generating off-centered focus, which can be regarded as an intrinsic displacement of the focus. In addition, we intentionally introduced spatially-varying beam-steering phases with a concentric shape, as in Figure 1(b). This spatially-varying beam-steerer is designed to generate discrete tangential wavevectors depending on the position of the incident beam on the second metasurface. The extrinsic displacement generated by the spatially-varying beam steerer visually instructs whether one should move the second metasurface in the vertical or lateral direction to get the best alignment. Only when two metasurfaces are well aligned, there will be no extrinsic discrete displacement. Consequently, the proposed device can support rotational-, longitudinal-, and transversal-alignment all at once with visual holograms.

With all functionalities considered, metasurfaces were designed based on the Jones matrix (see Methods for the details) and fabricated following the fabrication process described in the previous section. Figure 6(b)–(f) show the measured results of the built-in hologram. Note that this measured sample is the sample measured for the metalens doublet results in the previous section. Figure 6(b) shows the twin arrow image when it is centrally aligned with *θ*_*i*_(*i* = 1, 2) being zeros. Note that it shows clearly visible, right-directed, vertically separated and centered twin arrows image. Figure 6(c) shows the image when two metasurfaces are longitudinally misaligned. A deviation of only 10% of the target distance shows significant degradation of image quality. Figure 6(d) shows the image when the second metasurface is moved vertically. Note that while misalignment in vertical direction is intrinsic, our hologram shows extrinsic horizontal misalignment that can help align two metasurfaces with a glance. Figure 6(e) and (f) show the image when the rotation angles of metasurfaces are given as $\left(\theta_{1},\theta_{2}\right)=\left(\frac{\pi }{2},0\right)$ and $\left(\theta_{1},\theta_{2}\right)=\left(0,\frac{\pi }{2}\right)$ , respectively. All results verify that the device is working as expected.

As an application, we note that the given polarization-multiplexed metalens doublet and alignment-guiding hologram work simultaneously in a harmonious manner to realize a real-time-monitored three-dimensional focusing device. If the incident polarization is neither RCP nor LCP, but a mixture of both polarization states (i.e. elliptical polarization state), a portion of RCP is utilized for focusing and the remaining portion of LCP is used for monitoring based on the holographic image. Thus, if both devices are divided to take different optical paths at the output using optics (for example, a quarter waveplate and a polarization-dependent beam splitter), a real-time-monitored three-dimensional focusing device can be realized.

## 3 Conclusions

We have suggested a novel type of three-dimensional reconfigurable focusing device. With the device based on two beam-steering metalenses, we have theoretically, numerically and experimentally demonstrated that focus can be modulated in three-dimensional space by simply modulating their separation and rotation angles. Designed metasurfaces were fabricated based on a well-established lithography process and measured. In addition, by fully exploiting the potential of metasurfaces, we have designed polarization-multiplexed devices that perform as metalens doublet for RCP input and a visual alignment-guiding hologram for LCP input. Although we have proven the proposed scheme in near-infrared wavelength range, it can be easily extended to other wavelength ranges where dielectric metasurfaces based on unitary symmetric Jones matrices are already widely used such as THz [39], visible [35], or even ultraviolet [40] wavelengths. We anticipate that the simplicity of the tunable mechanism and the versatility of achievable functionalities that our proposed metalens doublet has shown can be widely applied to a variety of research fields such as three-dimensional imaging (a potential implementation incorporating a single-pixel photodetector illustrated in Supplementary Materials, Figure S10), facial recognition, laser cutting, and distance sensing, to name a few. Furthermore, while the proposed metalens doublet system consists of two passive metasurfaces in this study, the use of active metasurfaces may eschew the need for mechanical actuation, potentially broadening its applicability.

## 4 Methods

### 4.1 Unit cell simulation

To obtain transmission coefficient of nanopost structures with respect to their structural parameters, we employed the finite-difference time-domain (FDTD) commercial software from Ansys Lumerical Inc. A unit cell consists of an amorphous silicon (refractive index of 3.61 + 0.0055i at 915 nm) nanopost with an elliptical cross-section and a fused quartz substrate (refractive index of 1.45 at 915 nm), forming a square array with a period of 500 nm. The vertical dimension of silicon nanoposts were set to be 790 nm. Lateral dimensions along the major and minor axes were scanned from 100 nm to 300 nm considering experimental feasibility. For PI and PS devices, two groups with six elements each were chosen for the best performance corresponding to $\phi_{\text{M}}=\left\{\frac{3\pi }{12},\frac{7\pi }{12},\frac{11\pi }{12},\frac{15\pi }{12},\frac{19\pi }{12},\frac{23\pi }{12}\right\}$ , *ϕ*_m_ = *ϕ*_M_ for PI devices and *ϕ*_m_ = *ϕ*_M_ + π for PS devices.

### 4.2 Design of metasurfaces

For highly transmissive dielectric metasurfaces, based on the unitary symmetric Jones matrix approximation, the local response of the metasurfaces can be characterized with three parameters: phase accumulations of the principal axes *ϕ*_M/n_ and the rotation angle *θ*. For polarization-multiplexed metasurfaces, the ideal unitary symmetric Jones matrix is given as follows:

(4) $$\begin{array}{c} J\left(x,y\right)=\left[\begin{array}{cc} \left|w_{1}\right.\rangle & \left|w_{2}\right. \end{array}\rangle \right]\left[\begin{array}{cc} {\text{e}}^{\text{i}\phi_{1}\left(x,y\right)} & 0\\ 0 & {\text{e}}^{\text{i}\phi_{2}\left(x,y\right)} \end{array}\right]{\left[\begin{array}{cc} \left|v_{1}\rangle \right. & \left|v_{2}\right. \end{array}\rangle \right]}^{†} \end{array}$$

where $V=\left[\begin{array}{cc} \left|v_{1}\rangle \right. & \left|v_{2}\rangle \right. \end{array}\right]$ is an input orthogonal polarization pair, $W=\left[\begin{array}{cc} \left|w_{1}\rangle \right. & \left|w_{2}\rangle \right. \end{array}\right]$ is an output orthogonal polarization pair, $\phi_{1}\left(x,y\right)$ is the phase distribution of the first phase-only device through the first polarization channel (converted from $\left|v_{1}\rangle \right.$ to $\left|w_{1}\rangle \right.$ ) and $\phi_{2}\left(x,y\right)$ is the phase distribution of the second phase-only device through the second polarization channel (converted from $\left|v_{1}\rangle \right.$ to $\left|w_{1}\rangle \right.$ ). For PI design, the Jones matrix is obtained with *W* = *V* and *ϕ*_1_ = *ϕ*_2_. For PS design, the Jones matrix is obtained with $W=[\begin{array}{cc} \left|R\rangle \right. & \left|L\rangle \right. \end{array}]$ and $V=[\begin{array}{cc} \left|L\rangle \right. & \left|R\rangle \right. \end{array}]$ . To design real metasurfaces, three parameters at every location of metasurfaces are chosen to generate a Jones matrix being as close as possible to the ideal matrix. Since the proposed devices consist of two layers of metasurfaces, we used two polarization channels as follows: in the first channel, polarization states from incident $\left|R\rangle \right.$ to intermediate $\left|L\rangle \right.$ between metasurfaces to output $\left|R\rangle \right.$ , and in the second channel, polarization states from incident $\left|L\rangle \right.$ to intermediate $\left|R\rangle \right.$ between metasurfaces to output $\left|L\rangle \right.$ .

When designing the metasurfaces, the phase distribution of phase-only devices should be specified. The analytical phase distribution of the lens and beam-steering along the *x*-direction is widely known: $\phi_{\text{convex}}=-\frac{2\pi }{\lambda }\sqrt{x^{2}+y^{2}+f^{2}}$ , $\phi_{\text{concave}}=\frac{2\pi }{\lambda }\sqrt{x^{2}+y^{2}+f^{2}}$ and *ϕ*_steering_ = *k*_‖_ *x* where *f* is the focal length of a lens, λ is the wavelength, *ϕ*_convex_(*ϕ*_concave_) is the phase distribution of a convex(concave) lens, *k*_‖_ is the tangential wavenumber of beam-steering and *ϕ*_sttering_ is the phase distribution of a beam-steerer. For a spatially-varying beam-steerer, each concentric circle or circular ring in Figure 1(b) was designed to have gradually increasing tangential wavevectors in a stepwise manner from zero (the outer ring has the larger tangential wavevector). For a phase grating with a period Λ = 40 μm and a duty cycle of 0.5 along the *y*-direction, phase distribution of the grating is given as $\phi_{\text{grating}}=\overset{\infty }{\underset{n=-\infty }{\sum }}\Pi \left(x-n\Lambda \right)$ where $\Pi \left(x\right)=\left\{\begin{array}{c} 0,\;if\left|x\right|>\Lambda /2\;\\ \pi ,\;if\left|x\right|\leq \Lambda /2\; \end{array}\right.$ . For phase-only hologram design, the Gerchberg–Saxton (GS) algorithm was employed [41].

### 4.3 Optical characterization

The fabricated devices were characterized using the setup shown in Figure S7. The laser source with a wavelength of 915 nm was passed through a polarization controller, which consists of a half waveplate and a quarter waveplate to generate the desired incident polarization state. The size of the input beam was set to be 250 μm. After passing through the metalens doublet with finely tuned angles (*θ*_1_ and *θ*_2_) and separation *d*, the image of the plane right after the second metasurface can be transferred to a charge-coupled device (CCD) camera (CS505MU, Thorlabs) by a lens assembly consisting of an objective lens (MPLFLN 10x, Olympus) and a plano-convex lens (LSB04, Thorlabs). For measuring the performance of the metalens doublet, the assembly was set to form a 4-*f* system satisfying *s* = *f*. For measuring a holographic image, the assembly was set to satisfy *s* = 2*f* so that an image of the back focal plane (BFL) can be captured at the CCD plane.

## Supplementary Material

Supplementary Material Details
