# Supplementary material for: Three-dimensionally reconfigurable focusing of laser by mechanically tunable metalens doublet with built-in holograms for alignment
Source: Nanophotonics. 2023 Jan 16;12(8):1373–85. doi: 10.1515/nanoph-2022-0634 (PMC11501995; doi:10.1515/nanoph-2022-0634)
Supplement: Supplementary file 1 — Supplementary Material Details [file j_nanoph-2022-0634_suppl.pdf]

## Supplementary Materials for "Three-dimensionally reconfigurable focusing of laser by mechanically tunable metalens doublet with built-in hologram for alignment"

*Joonkyo Jung*[1,†], *Hyeonhee Kim*[1,†] and *Jonghwa Shin*[1,*]

[1]Department of Materials Science and Engineering, KAIST, Daejeon 34141, Republic of Korea.

[†]These authors equally contributed to this work.

[*]E-mail: qubit@kaist.ac.kr

### Supplementary texts

### S1. The Fresnel approximation of metalens doublet

We analyzed metalens doublet using the Fresnel approximation to obtain the analytical equation of location of the resulting focus. For that purpose, first, the Fresnel approximation of each lens are derived, separately. Then, they are combined to obtain the final closed form equation of location of the focus.

Figure S1(a) shows the first beam-steering metalens (BSML) configuration. We assumed plane wave incidence ($f(x',y')=1$) rather than the Gaussian beam for simplicity. The first convex BSML has focal length of $f_1$ and tangential wavevector of $\boldsymbol{k}_{\parallel,1}=\hat{\boldsymbol{x}}k\delta_{x,1}+\hat{\boldsymbol{y}}k\delta_{y,1}$ where $\hat{\boldsymbol{x}}$ and $\hat{\boldsymbol{y}}$ are unit vectors and $k$ is free space wavenumber; in other words, the phase distribution is given as $\phi_{\mathrm{BSM\,L,1}}=\left(-k\sqrt{x'^2+y'^2+f_1^2}\right)+\left(k\delta_{x,1}x'+k\delta_{y,1}y'\right)$. In this case, the output field profile $g(x,y)$ is derived as follows:

$$g(x,y)=\left(\frac{1}{i\lambda f_1}\right)\exp(ik f_1)\iint f(x',y')\exp\left(i\phi_{\mathrm{BSM\,L,1}}\right)\exp\left[i\pi\frac{(x-x')^2+(y-y')^2}{\lambda f_1}\right]dx'dy'$$
$$\approx\left(\frac{1}{i\lambda f_1}\right)\exp\left[\frac{i\pi(x^2+y^2)}{\lambda f_1}\right]\iint\exp\left[-i2\pi\left\{x'\left(\frac{2x}{2\lambda f_1}-\frac{2\delta_{x,1}}{2\lambda}\right)+y'\left(\frac{2y}{2\lambda f_1}-\frac{2\delta_{y,1}}{2\lambda}\right)\right\}\right]dx'dy'$$
$$=\left(\frac{1}{i\lambda f_1}\right)\exp\left[\frac{i\pi(x^2+y^2)}{\lambda f_1}\right](\lambda f_1)^2\delta_D\left(x-f_1\delta_{x,1}\right)\delta_D\left(y-f_1\delta_{y,1}\right)$$
$$\therefore g(x,y)\propto\delta_{\mathrm{D}}\left(x-f_1\delta_{x,1}\right)\delta_{\mathrm{D}}\left(y-f_1\delta_{y,1}\right)$$

where $\delta_D$ is the Dirac delta function. We used the Taylor expansion of $\left(x'^2+y'^2+f_1^2\right)^{1/2}=\approx f_1\left(1+\frac{x'^2+y'^2}{2f_1^2}\right)$ assuming that the focal length is much larger than the sample dimensions.

Figure S2(b) shows the second beam-steering metalens configuration. The second concave BSML has focal length of $f_2$ and tangential wavevector of $\boldsymbol{k}_{\parallel,2}=\hat{\boldsymbol{x}}k_0\delta_{x,2}+\hat{\boldsymbol{y}}k_0\delta_{y,2}$, in other words, the phase distribution is given as $\phi_{\mathrm{BSM\,L,2}}=\left(k\sqrt{x'^2+y'^2+f_2^2}\right)+\left(k\delta_{x,2}x'+k\delta_{y,2}y'\right)$. Given $g(x',y')$ being the focused image of the first BSML, the output field profile $m(x,y)$ is derived :

$$h(x',y') = \left(\frac{1}{i\lambda\,(-d_1)}\right)\exp\left(ik\,(-d_1)\right)\iint g(x',y')\exp\left[i\pi\,\frac{(x'-x')^2+(y'-y')^2}{\lambda(-d_1)}\right]dx'\,dy'$$

$$m(x,y) = \left(\frac{1}{i\lambda\,d_2}\right)\exp(ik\,d_2)\iint h(x',y')\exp\left(i\phi_{\mathrm{BSM\,L,2}}\right)\exp\left[i\pi\,\frac{(x-x')^2+(y-y')^2}{\lambda d_2}\right]dx'dy'$$

$$\approx \frac{d_1}{d_2}\exp[-ik\,(f_2+d_1-d_2)]\exp\left[\frac{i\pi\,(x^2+y^2)}{\lambda f_2}\right]\exp\left[\frac{i\pi}{\lambda f_2}\left\{\left(\frac{d_1x}{d_2}-d_1\delta_{x,2}\right)^2 + \left(\frac{d_1y}{d_2}-d_1\delta_{y,2}\right)^2\right\}\right]g\left(\frac{d_1x}{d_2}-d_1\delta_{x,2},\frac{d_1y}{d_2}-d_1\delta_{y,2}\right)$$

$$\therefore m(x,y) \propto g\left(\frac{d_1x}{d_2}-d_1\delta_{x,2},\frac{d_1y}{d_2}-d_1\delta_{y,2}\right)$$

where $h(x',y')$ is the field profile right before the second metalens obtained by considering free space propagation of $g(x',y')$. By combining two equations, one can directly obtain the following:

$$m(\boldsymbol{\rho}) \propto \delta\left[\boldsymbol{\rho} - \frac{d_2}{k_0}\left(\boldsymbol{k}_{\parallel,2} + \frac{f_1}{d_1}\boldsymbol{k}_{\parallel,1}\right)\right].$$

From the above equation, Eq. (1) in the manuscript can be obtained.

## S2. Angled incidence condition for the uninterrupted region at the center

As explained in the manuscript, the achievable region has doughnut-like shape under the normal incidence condition. On the contrary, if the incident beam is tilted, the uninterrupted region can be centered. This result can be directly derived from the Fresnel approximation in the previous section. Under the angled incidence condition, the transversal displacement $\boldsymbol{\rho}'$ is given as

$$\boldsymbol{\rho}' = \frac{d_2}{k_0}\left(\boldsymbol{k}_{\parallel,2} + \frac{f_1}{d_1}\boldsymbol{k}_{\parallel,1} + \frac{f_1}{d_1}\boldsymbol{k}_{\parallel,\mathrm{inc}}\right) = \boldsymbol{\rho} + \boldsymbol{\rho}_{\mathrm{inc}}$$

where $\boldsymbol{k}_{\parallel,\mathrm{inc}}$ is a tangential wavevector of the incident beam, $\boldsymbol{\rho}$ is the transversal displacement under the normal incidence condition and $\boldsymbol{\rho}_{\mathrm{inc}}$ is a newly added displacement by the angled incidence. Note that $\boldsymbol{\rho}_{\mathrm{inc}}$, which comes from the angled incidence, is a constant term regardless of rotation angles of metasurfaces. This constant term effectively shifts the achievable region defined by $\boldsymbol{\rho}$ translationally. The effect of this shift on the achievable region is compared in Figures S3(a,b).

## S3. Spectral analysis of fabrication error

To qualitatively understand amplitude degradation generated by errors in pattern size or sidewall slope, we conducted spectral analysis of nanostructures. We employed the FDTD simulation to obtain transmission spectrum of four circular post structures with radius of {181 nm, 190 nm, 200 nm, 204 nm} as in Figure S6. As the radius of post increases, resonance wavelength of transmission dip, which is marked with a blue circle, also increases, as explained in the manuscript. In particular, the post with radius of 200 nm shows strongly suppressed transmission at the target wavelength of 915 nm. These results can explain why the third and fifth unit cell structure for PI design shows significant degradation in transmission for positive

errors in pattern size or sidewall slop and the fourth structure shows significant degradation in transmission for negative errors as in Figure 4 and Figure S5.

**S4. Paths of polarization conversion**

Ideally, for the polarization-sensitive design, each metasurface has to perfectly convert the input circularly polarized waves into the circular polarization state with the opposite handedness with desired phase responses as depicted in Figure S8(a). As a result, if the conversion efficiency is unity, i.e. 100%, the output polarization states are going to be the same as the input polarization states because of double polarization conversions (e.g., RCP to LCP to RCP). However, if the conversion efficiency does not reach 100% due to fabrication or other errors, there will be a residual component that has the same polarization states as the input states after passing through a metasurface. Let us consider RCP input polarization state as an example. In this case, since we have two metasurfaces, there are four paths that polarization states can be converted as depicted in Figure S8(b): (1) RCP → LCP → RCP, correctly converted two times; (2) RCP → RCP → LCP and (3) RCP → LCP → LCP, correctly converted only one time, and incorrectly converted the other time; (4) RCP → RCP → RCP, incorrectly converted two times. Only the first path is the desired one, and the other three terms contribute to noise. One thing to note is that the second and third paths, which are first-order noises, result in LCP output polarization state and can be easily eliminated by inserting a polarization filter or polarizing beam splitter. The fourth term is a second-order noise term and usually much weaker than the other components. For LCP input polarization state, the above discussion can be applied in the same way.

**Supplementary figures and tables**

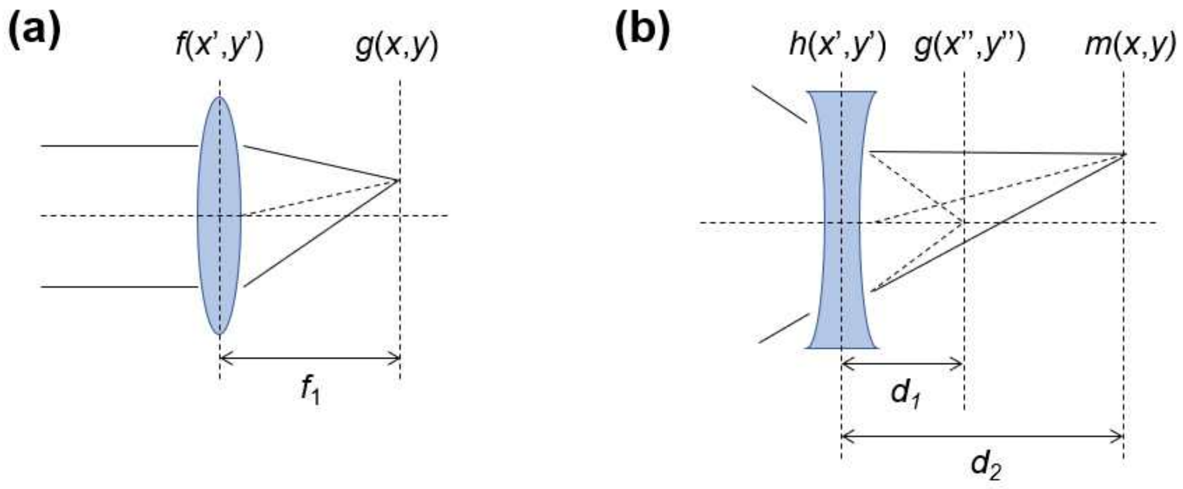


**Figure S1. The Fresnel approximation of metalens doublet.** (a) Configuration of beam-steering convex lens with focal length $f_1$ and beam-steering tangential wavevector $\boldsymbol{k}_{||,1}$. (b) Configuration of beam-steering concave lens with focal length $f_2$ and beam-steering tangential wavevector $\boldsymbol{k}_{||,2}$.

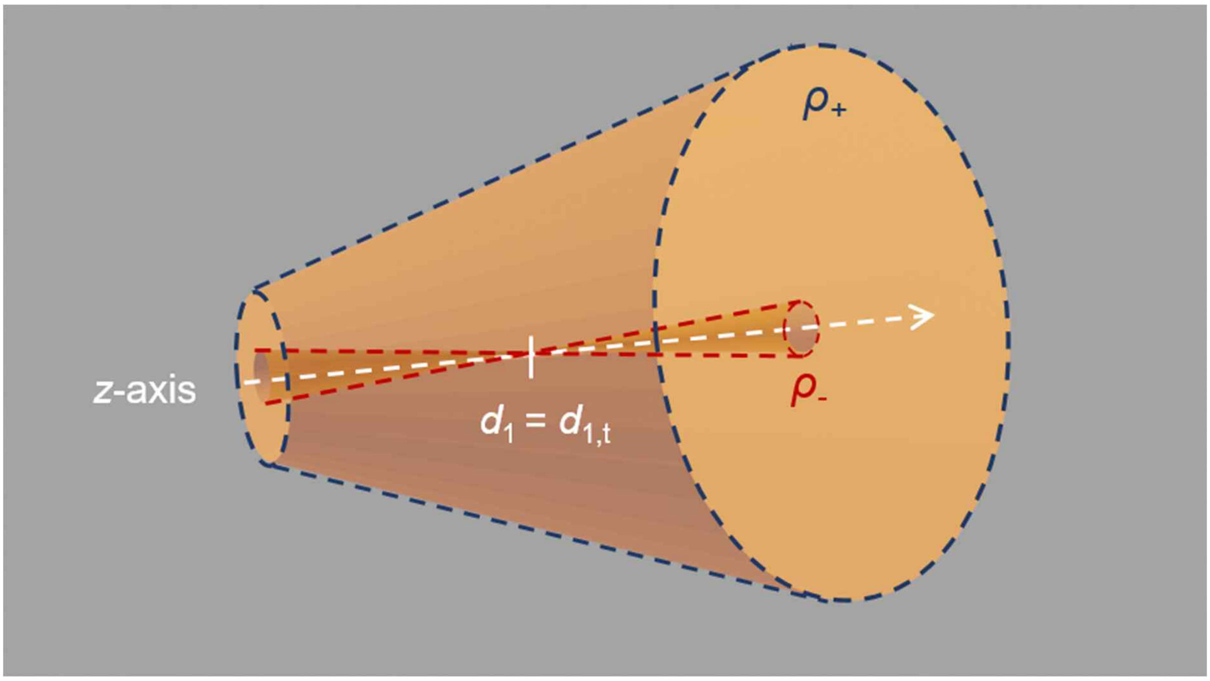


**Figure S2. Three-dimensional illustration of the achievable region of tunable hot spot.** White dotted line shows *z*-axis. Blue(red) dotted line represents layout of the maximal(minimal) displacement of $\rho_+(\rho_-)$. All displacement between the maximal and minimal values (i.e. between red and blue layout) can be achieved. The hollow space around the *z*-axis is the theoretically forbidden region. Note that the center point can be achieved when $d_1 = d_{1,\mathrm{t}}$.

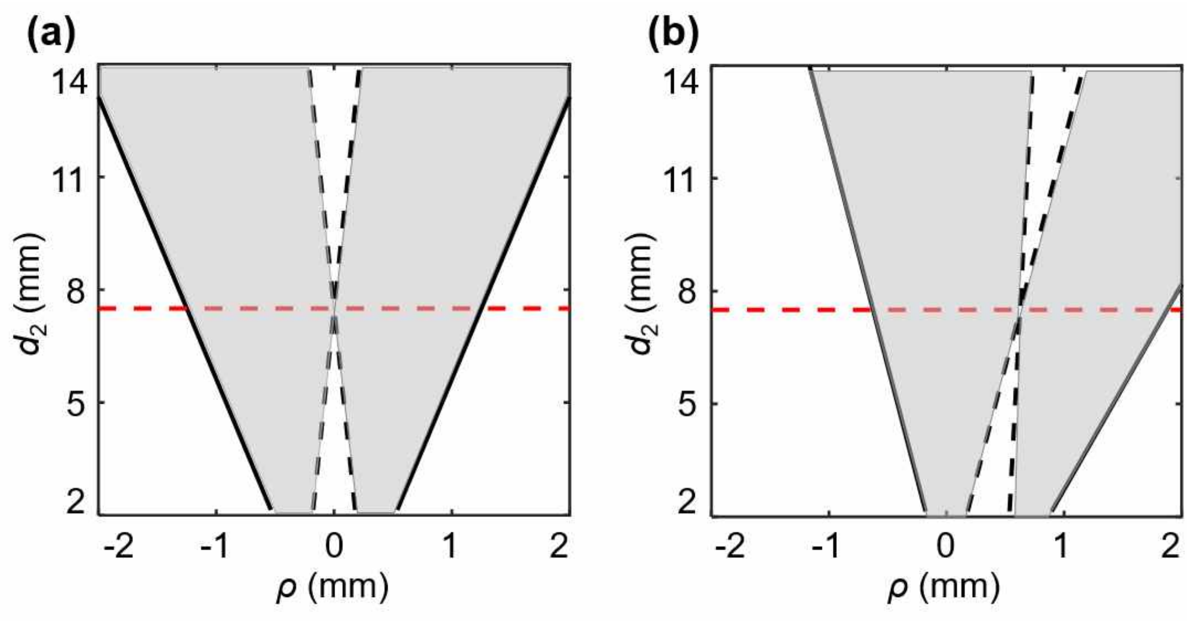


**Figure S3. Effect of angled incidence for the uninterrupted region at the center.** (a) Numerical demonstration of achievable region under the normal incidence condition ($|\boldsymbol{k}_{||,\text{inc}}|= 0$). Black dashed(solid) lines shows the minimal(maximal) displacement. (b) Numerical demonstration of achievable region under the angled incidence condition ($|\boldsymbol{k}_{||,\text{inc}}| = |\boldsymbol{k}_{||,1}|$). Black dashed(solid) lines shows the corrected minimal(maximal) displacement under the angled incidence condition. Gray shaded regions are where the focus can be placed by manipulating rotation angles of metasurfaces. Assumed parameter are as follows: $|\boldsymbol{k}_{||,1}| = 0.05k_0$, $k_0 = 2\pi/\lambda$, $\lambda = 915$ nm, $d_{1,\text{t}} = 3$ mm, $f_1 = 5$ mm, $f_2 = -5$ mm.

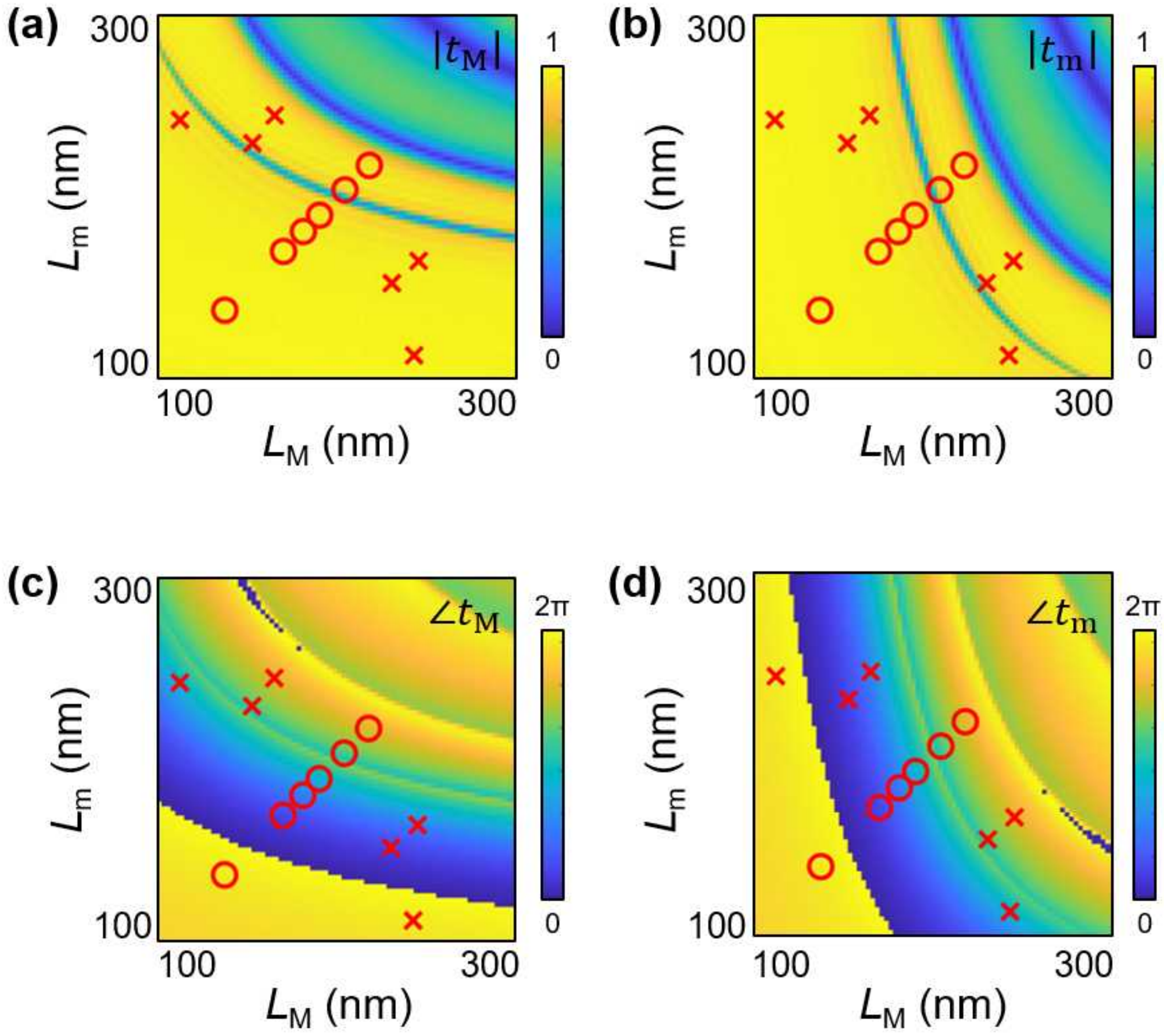


**Figure S4. Graphical representation of transmission coefficient of unit cell.** (a) Phase of transmission coefficient along the major axis. (b) Phase of transmission coefficient along the minor axis. (c) Magnitude of transmission coefficient along the major axis. (d) Magnitude of transmission coefficient along the minor axis. Red circles(crosses) represent the selected structure for the PI(PS) design.

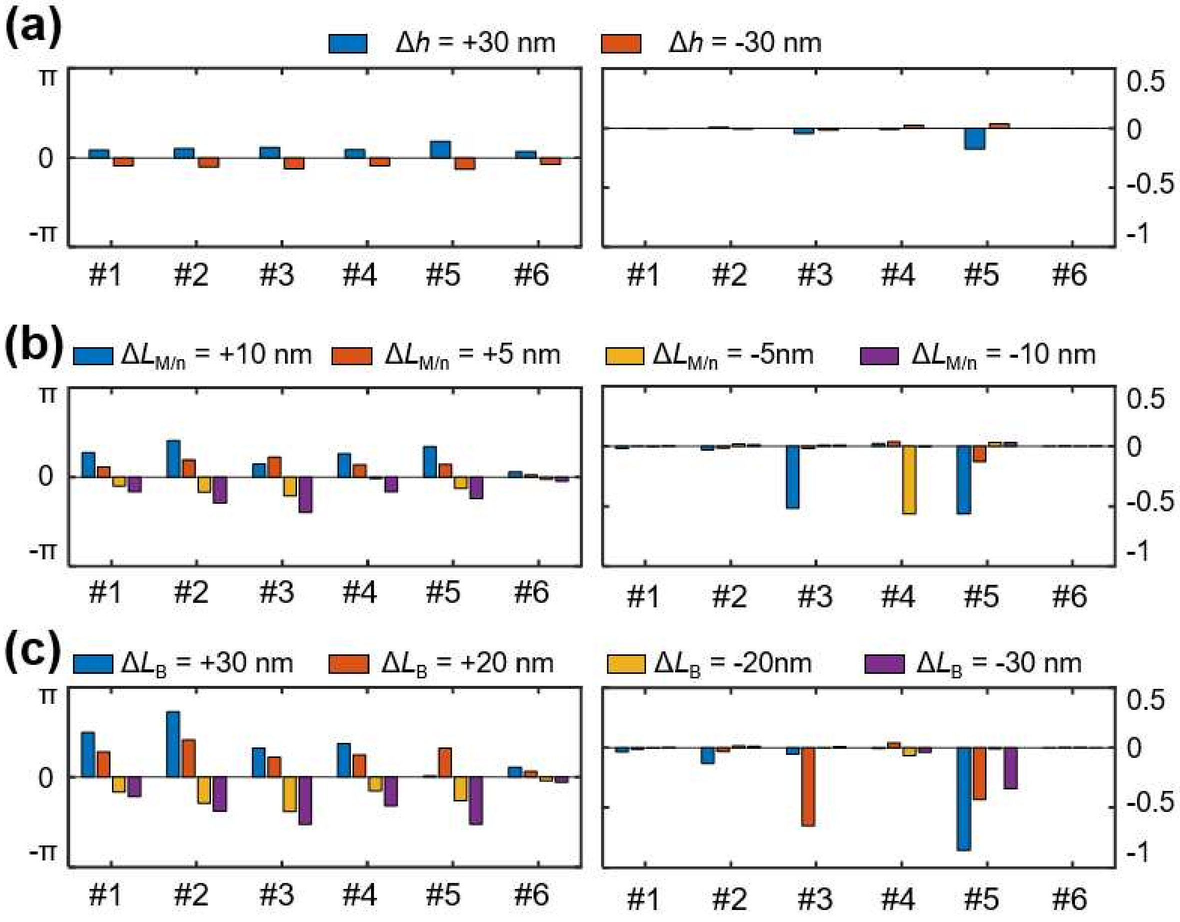


**Figure S5. Performance degradation of the selected design for the PI design.** (a-c) Phase(amplitude) deviation from the target value for the selected structure of the PS design on left(right) panel. (a) Errors in thickness, $\Delta h$. (b) Errors in pattern size along the major and minor axes, $\Delta L_{M/m}$. (c) Errors in sidewall. Bottom cross-section is larger or smaller compared to top cross-section by amount of $\Delta L_B$.

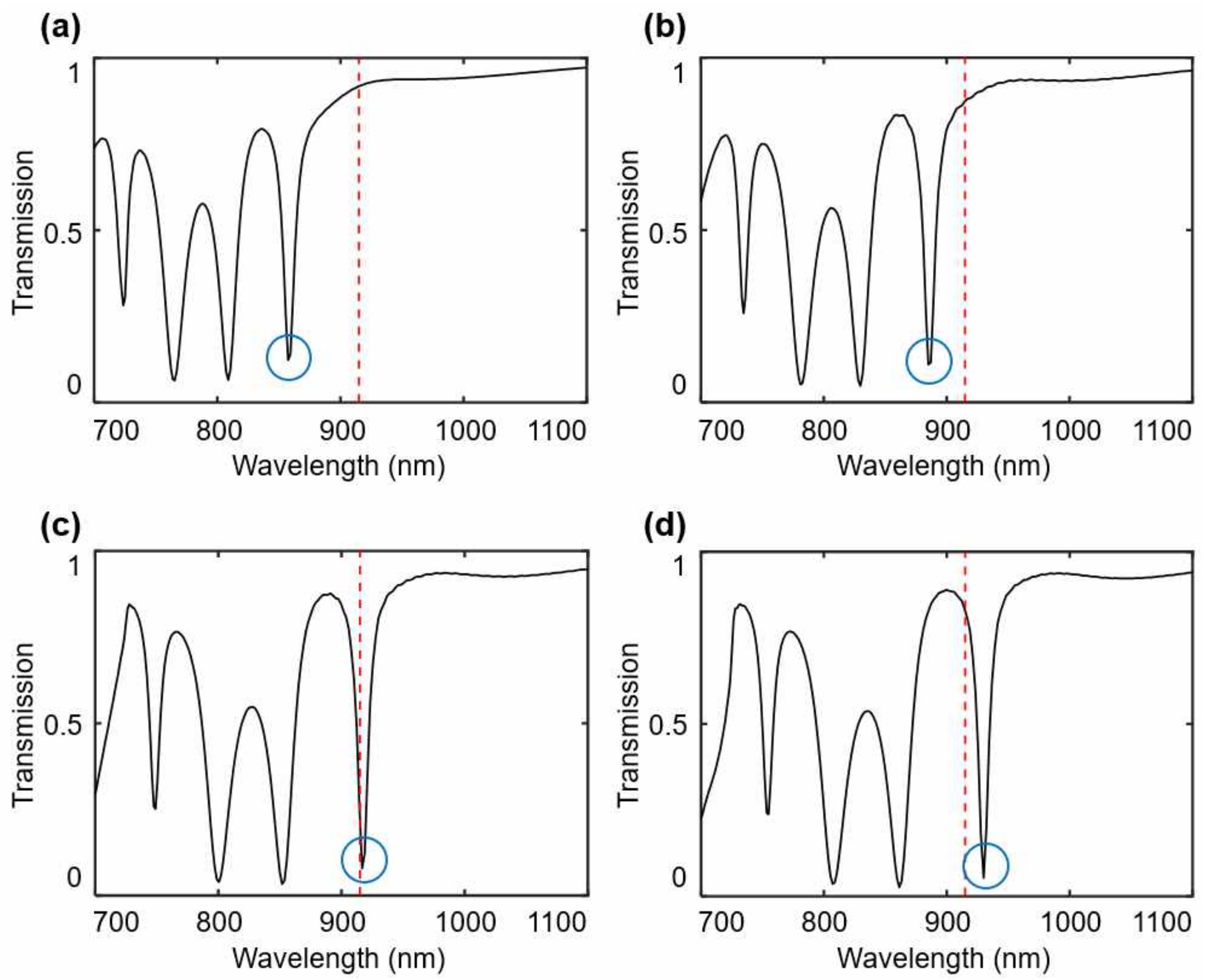


**Figure S6. Spectral analysis of unit cell.** (a) Transmission spectrum of circular post with radius of 181 nm. (b) Transmission spectrum of circular post with radius of 190 nm. (c) Transmission spectrum of circular post with radius of 200 nm. (d) Transmission spectrum of circular post with radius of 204 nm. Blue circles show the resonance wavelength of transmission dip increases as the radius of post increases. Red dashed line shows target wavelength of 915 nm.

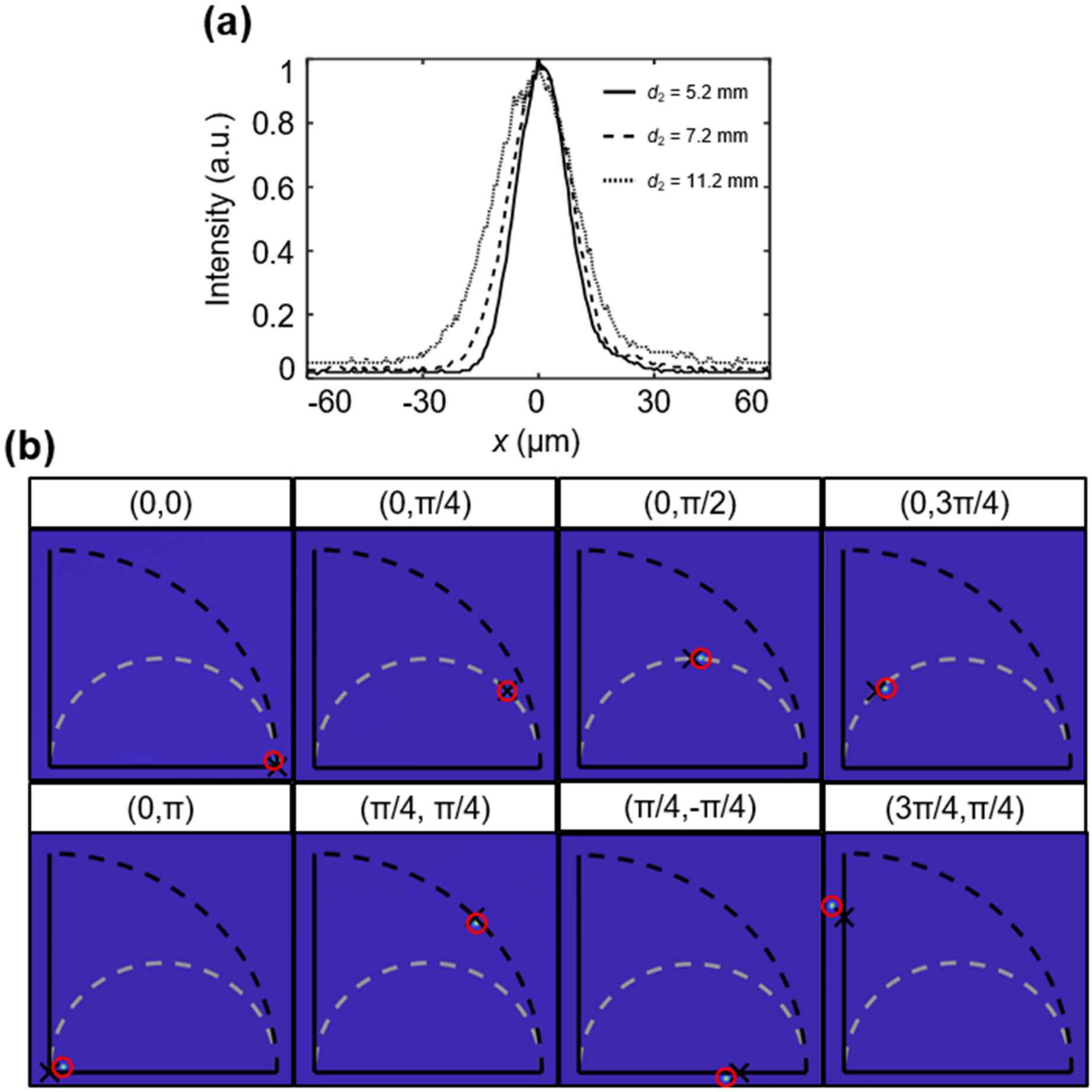


**Figure S7. Measurement of tunable metalens doublet with PI design.** (a) Experimentally measured intensities at different $d_2$ value. (b) Measured field profiles for various combinations of rotation angles of metasurfaces

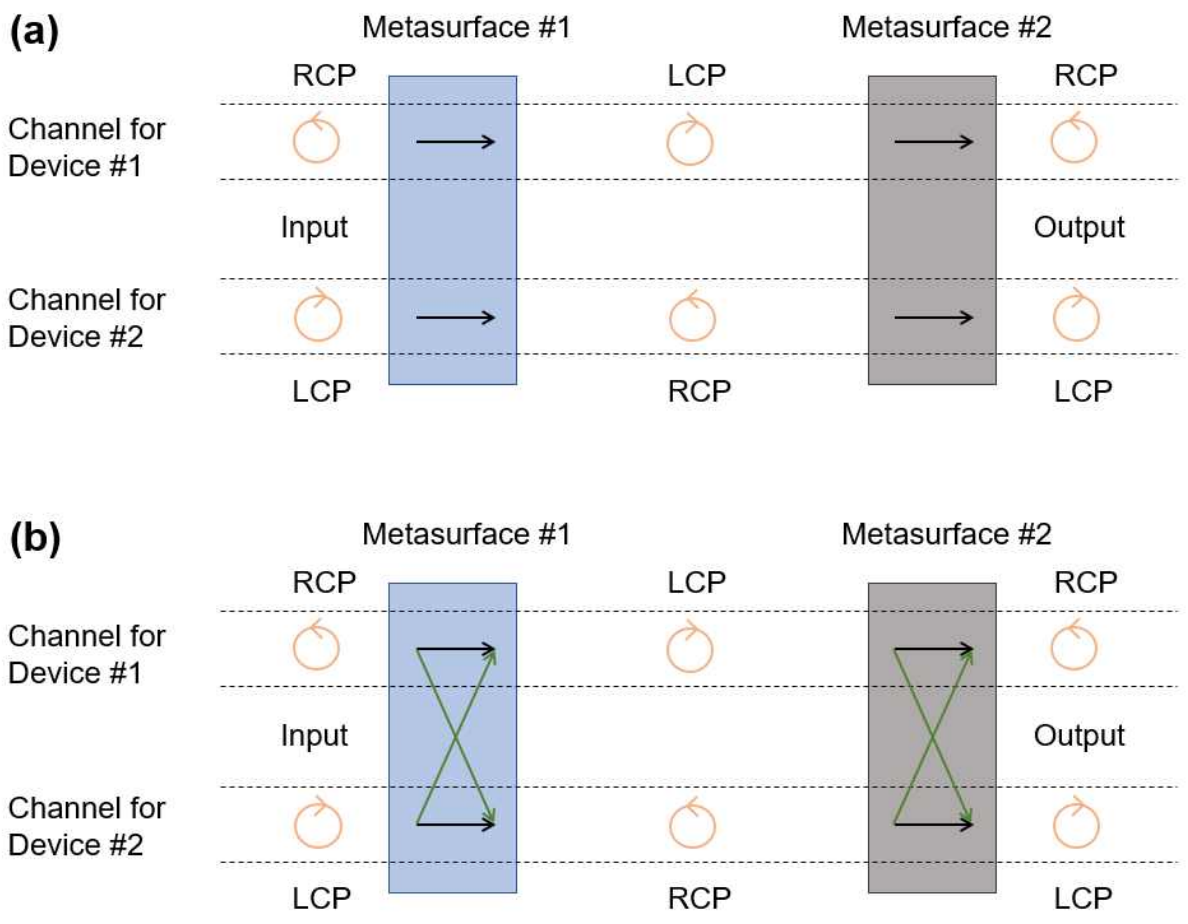


**Figure S8. Path of polarization conversion.** (a) Ideal polarization channels with unity conversion efficiency. Each metasurface perfectly converts the input RCP/LCP into the output LCP/RCP polarization states. Each polarization channel is perfectly separated. Orange circles represent polarization ellipses. Black arrows represent the desired polarization conversion. (b) Realistic polarization channels with non-unity conversion efficiency. Each metasurface converts the input RCP/LCP into the output LCP/RCP polarization states with a small amount of residual unconverted energy, which has the same polarization states with the incident light. Orange circles represent polarization states. Black arrows represent the desired polarization conversion. Green arrows represent the undesired polarization conversion, which contribute to noise of the system.

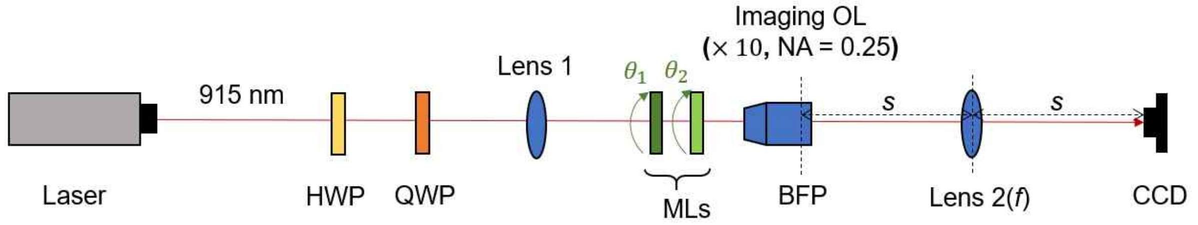


**Figure S9. Optical measurement setup.** HWP: half waveplate; QWP: quarter waveplate; ML: metalens; OL: objective lens; BFP: back focal plane; CCD: charge-coupled device.

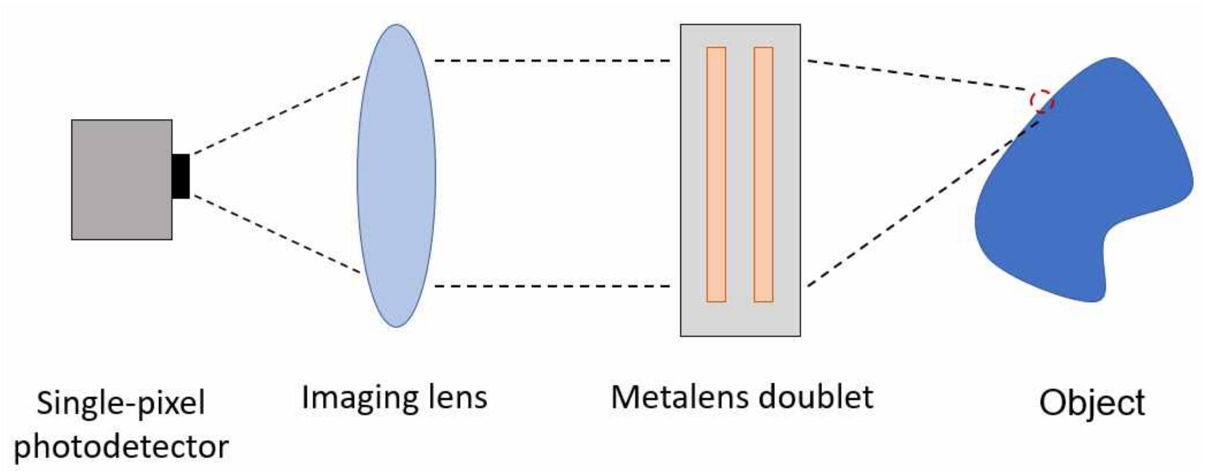


**Figure S10. Three-dimensional point-by-point imaging device.** The suggested device is designed to obtain three-dimensional images by measuring the local intensity of an object. Single-pixel photodetector measure the intensity from the target point which is marked with the dotted red circle. By utilizing the tunability of the metalens doublet, the measured point on the object can be tuned at will.

## Table S1. Efficiencies of devices with fabrication errors

| Error type | Hologram (PS) | Lens (PS) | Hologram (PI) | Lens (PI) |
|---|---|---|---|---|
| Ideal | 80.37% | 82.47% | 79.62% | 81.70% |
| Thickness (+30 nm) | 76.68% | 78.66% | 72.79% | 74.69% |
| Thickness (-30 nm) | 80.30% | 82.42% | 80.39% | 82.54% |
| Pattern (+10 nm) | 62.53% | 64.34% | 45.96% | 47.40% |
| Pattern (+5 nm) | 76.46% | 78.41% | 73.72% | 75.60% |
| Pattern (-5 nm) | 65.54% | 67.25% | 63.22% | 64.89% |
| Pattern (-10 nm) | 73.21% | 75.34% | 72.42% | 74.52% |
| Sidewall (+30 nm) | 37.91% | 39.34% | 35.37% | 36.85% |
| Sidewall (+20 nm) | 57.96% | 59.66% | 46.65% | 48.04% |
| Sidewall (-20 nm) | 73.09% | 75.16% | 69.39% | 71.37% |
| Sidewall (-30 nm) | 60.06% | 61.87% | 53.87% | 55.51% |
